# The role of maternal trauma and discipline types in emotional processing among Syrian refugee children

**DOI:** 10.1007/s00787-022-01962-3

**Published:** 2022-02-26

**Authors:** Kirsi Peltonen, Gustaf Gredebäck, Seth D. Pollak, Marcus Lindskog, Jonathan Hall

**Affiliations:** 1 Department of Child Psychiatry and INVEST Research Flagship Center, University of Turku, Lemminkäisenkatu 3, 20014 Turku, Finland; 2 Uppsala University, Uppsala, Sweden; 3 University of Wisconsin-Madison, Madison, USA

**Keywords:** PTS, War, Emotion, Parenting, Discipline, Face recognition

## Abstract

Stressful experiences in armed conflict incur intergenerational effects through parental behaviors with their children. A recent study reported that among Syrian refugee families, mothers’ (but not fathers’) post-traumatic stress (PTS) impacted children’s emotional processing. In this study, we aim to shed further light on this phenomenon by analyzing how the parenting practices in the context of post-traumatic stress confers protection or risk for children’s emotional processing. Participants were 6–18-year-old children (*n* = 212) and their mothers (*n* = 94), who fled from Syria and were residing in Turkish communities. We used the computer-based emotional processing task including photos of facial movements typically associated with different emotions to measure children’s capacity for emotional processing. Mothers reported their PTS and the discipline types they use, as well as the contextual factors related to their refugee background. Linear mixed effect models were constructed first, to find out the discipline types that are most strongly associated with emotional processing of the child, and second, to examine whether these discipline types moderate the effect of maternal PTS on children's emotional processing. Finally, generalized linear models were constructed to examine which contextual factors are associated with the use of these discipline types by mothers. We found that spanking as a discipline type was associated with poorer child emotional processing, whereas withholding of media access was associated with better emotional processing. Younger and less religious mothers were more prone to use spanking. The study underlines the need for parenting programs alongside with efforts to address mental health issues among mothers living under armed conflict.

**Supplementary Information:**

The online version contains supplementary material available at 10.1007/s00787-022-01962-3.

## Introduction

Parenting in war and displacement is especially challenging. Parents who are themselves dealing with various forms of adversity are also facing substantial difficulties tending to the needs of their children, including a shortage of resources, perceived chaos in their daily lives, and lack of safety [1, 2]. In these circumstances, parents often observe negative changes in children’s behavior and tend to adopt negative and harsh parenting styles and discipline types to meet these changes [1]. The meta-analysis of Eltanamly et al. [3] showed that parents with more exposure to war time atrocities showed less warmth and more harshness towards their children, which partly mediated the association between war exposure and child adjustment. However, some parents are able to maintain warm and positive parenting despite the stressful living conditions [4]. The extant literature suggests that severe stress and mental health problems of parents are likely to be associated with difficulties in children’s adjustment and behavior [5, 6].

Mothers serve as the primary caregivers of children in Middle-Eastern families, and especially the mother-related factors have been found to impact child development in war-affected areas [6, 7] Maternal mental health and caring are associated with relational problems and social withdrawal [8], psychiatric symptoms [9], sensorimotor and language development [10], and lower educational attainment [5] of a child. [11] showed that mothers’, but not fathers’, post-traumatic stress is related to children’s emotional processing. However, the bi-directional nature of adversity and family dynamics must be considered, as children’s emotional processing difficulties might challenge parent–child interaction, generate family conflicts and require additional parental resources. There might be higher level of conflicts in the family due to these difficulties and challenges in optimal parent–child interaction.

Emotional processing, the ability to make reasonable inferences based upon other people’s facial expressions is fundamental in early interpersonal communication. Later on, it also plays a prominent role in face-to-face interactions [12]. Emotional processing develops early in interactions during childhood [13, 14], and the parent–child relationship appears fundamental in this process [12]. Indeed, poor attachment quality in general has been found to lead to a broad deficit in emotional processing [15, 16], and exposure to anger and violence leads to a heightened sensitivity to angry faces [17, 18]. Notably, this work has to a large extent been carried out in Western societies and in peace time conditions.

In a recent exception to this Western-oriented research, [11] showed that among Syrian families living in Konya, Turkey, mothers’ post-traumatic stress impacted children’s emotional processing. Research with refugees have further demonstrated that stressful conditions, together with parental mental health problems, have a negative impact on the quality of parent–child interactions and attachment [6, 19]. In a broader context, the recent meta-analysis showed that early adversity (including many types of harmful experiences such as abuse, bullying, and social deprivation) relates to alterations in behavioral and neurophysiological processing of facial emotions [20]. However, when directly looking into parenting practices and/or discipline types on emotional processing of a child, no such effects can be found [21–23]. At face value, these results seem to contradict each other. On the one hand, parent’s mental health impacts the attachment relationship, parent–child interactions, and emotional processing in children; yet, no direct associations between parenting practices and emotional processing of a child have emerged.

One way to reconcile these diverse findings is to investigate if parental mental health and parenting practices interact. For example, in the context of war and refugees, some parental practices might, despite parent mental health problems, protect the child’s emotional development. Conversely, other forms of parenting might cause an additional risk for non-optimal development. If parenting practices such as Spanking or other adverse forms of discipline are frequent and related to emotional processing, it is important to know which factors are associated with the use of such discipline types. There might be something driving the parenting practices parents use. In this study, we investigate this possibility. Earlier research suggests that factors that may correlate with the forms of discipline used by parents may include socioeconomic status, education, religiousness, and experiences of own discrimination [24–26].

Given that the earlier findings have implicated the association between maternal mental health and emotional processing of the child, but not between the parenting practices and emotional processing of the child, we explored whether the interaction effects between maternal PTS and different discipline types exist. Since our study sample and methods are distinct from prior studies, we also tested whether the direct associations between the discipline types and child emotional processing competence emerged. The goal of this paper is to examine: (1) which discipline types are associated with children’s emotional processing competence; (2) whether these discipline types moderate the impact of maternal traumatic stress on child emotional processing (either by supporting or reducing emotional processing) and (3) which maternal factors are associated with the use of those discipline types that support the healthy development of children’s emotional processing. To accomplish this goal, we combine cross-sectional experimental and questionnaire data from 394 individuals (representing 100 Syrian refugee families) living in Turkish communities (the same dataset previously used [11]). We measure children’s emotional processing and relate it to mothers’ self-reports of war-related post-traumatic stress and discipline types. We use only the data from mothers since the earlier findings, using the same data, showed that only maternal (and not paternal) PTS had effect on child emotional processing, which is the starting point of our moderation analysis.

## Methods

### Participants

The participants were 212 children (*M*_age_ = 12.2, SD_age_ = 3.1, range = [6, 18] 42.3% girls) and their mothers (*n* = 94, *M*_age_ = 37, SD_age_ = 7.12, range = [22, 60]) in a total of 100 Syrian families living in Konya, Turkey. The median number of children in each family was 2, range = [1, 5]. Nearly, all families originate from Aleppo (*n* = 151), in addition to a few families from Ar Raqqah (*n* = 1), Damascus (*n* = 4), Deir al-Zour (*n* = 2), Homs (*n* = 1), Idlib (*n* = 4) and Lattakia (*n* = 6).

### Procedure and design

The recruitment of participants was based on an opportunistic sampling procedure. The records of refugee families with children in our target community setting was not available, and therefore, the fieldwork coordinator referred the families from his personal network. These families were then asked to recommend the study to other families. The data collection took place between October 2019 and January 2020. Syrian research assistants, who were fluent in Arabic and Turkish, visited the homes of these refugee families, and were supervised by a Turkish fieldwork coordinator. The study protocol lasted approximately 30 min for children and 60 min for adults over 18 years. Multiple family members participated in parallel, and sometimes children needed help from parents, which made the length of the session highly variable. Two researchers (one male, one female) supervised each session and parents helped children understand the tasks if needed. Each family received a monetary compensation equivalent of 10 Euro per participant for participation. Written and verbal informed consent from all participants were asked. The study was approved by the Necmettín Erbakan University in Turkey (2019/17) and regional ethics review board in Sweden (2018-395).

After receiving the consent for the study from the participating family members, the study started with tea and biscuits (brought by the research team). Following this, each family member sat in front of a computer (DELL Vostro 3568, 15’ screen). The participants wear noise-canceling headphones while completing a larger battery of experimental tasks. The tasks assessed IQ [27], visual attention [28, 29], emotional processing [30], working memory [31], and risk-taking [32] with written instructions in Arabic. The task was identical for all ages and all participants had the same amount of time to complete the task. After the experimental tasks, adult participants also completed a series of questionnaires (all in Arabic), which assessed the demographics, social environment, migration history, risk factors, discrimination, potentially traumatic events, and post-traumatic stress [33–37] (see Supplementary Information of [11] for complete list). The current paper reports on emotional processing of children as well as maternal post-traumatic stress and discipline types.

**Fig. 1:**
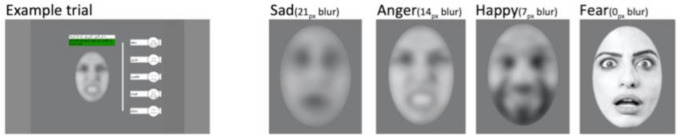
Stimuli used in the emotion processing task assessing social cognition. The text on the example trial can be translated as “How does this person feel? From the words on the right, click which one you think is best” [11]

The emotional processing task included photos of eight adult (4 female), Arab faces conveying facial movements typically associated with anger, happiness, sadness, fear, and a neutral expression. In earlier [11] study, the images were altered such that faces initially contained only partial information that became more clear over time (ranging from low-frequency images where the emotions were difficult to perceive to clear images that portrayed faces in full clarity, see Fig. 1), following the procedure of Pollak and Sinha [38] and modified by Forslund et al. [15]. To gain power in the analysis of the current study, the data were combined into one emotional recognition accuracy score by averaging the emotions scores across different emotions (sad, anger, happy, and fear). The final value thus represents the average of all emotions. Each image was rated by 37 university students at the Necmettín Erbakan University to confirm that the photos were easily categorized and discriminated (See SI for rating descriptives). Initial pilots from Uppsala and Konya with refugee children indicated that children of the target ages understood the task and responded correctly.

### Measures

#### Mothers stress

The Post-Traumatic Stress Questionnaire, PCL-C_Abbreviated_ [36] includes six questions related to disturbing memories, negative emotions related to past experiences, avoidance of situations that remind of stressful experiences, distance to other people, irritation and anger, and concentration difficulties. For each item, responses were provided on a 5-point scale (from *not at all* to *extremely*) asking how much they have been bothered by each problem in the last month. The dependent variable was the aggregate score ranging from 6 (not been bothered by any of these problems during the last month) to 30 points (extremely bothered by all problems during the last month).

Discipline types used by mother were measured by asking a parent the following question: sometimes children get so angry at their parents that they act out in a temper tantrum or do not do as they are told. Please indicate if you have ever used the following discipline types with your children when they act out or do not do as they are told (yes/no): Grounding; Spanking; Household Chores/Duties; Send child to his/her room for 1 h or more; Take away TV/Phone, Game or Activity privileges; Put your child in a short time out (less than 1 h); Talk with your child about his/her actions.

#### Social context

We measured multiple aspects of parents’ social context including changes in socioeconomic status, experiences of discrimination, education, religiosity, and urbanicity as these all might influence parent’s choice of discipline practices.

To measure downward mobility, we relied upon two self-reported measures of perceived socioeconomic status (SES), one for Turkey and one for participants’ country of origin prior to the war. These questions read as follows: “Imagine (Turkish society/the society in your country of origin) (Syria or Iraq) as arranged on a scale like the one shown below, where the worst off socially and economically are on the left (0) and the best off are on the right (10). Please move the slider to select the place where you stand/feel you stood prior to the war.” Downward mobility is measured as the difference between these two variables. For example, an individual with a score of 5 in their country of origin and 4 in Turkey would have a downward mobility score of 1.

To measure discrimination, we asked respondents if during the past 12 months they were every badly treated because of their foreign background. The Discrimination Index is an additive index of the following seven response categories: when looking for housing; when looking for work; in contacts regarding studies; in contacts regarding medical services; in contacts with other public authorities; during encounters in the street or in public transport; in other situations.

Education is a continuous measure representing the highest level of education the participant completed. The response categories included: I have no formal education; I have completed less than 6 years of schooling; I have completed 6 years of schooling; I have completed 9 years of schooling; I have completed 12 years of schooling; I have completed more than 12 years of schooling.

Religiosity was measured on a six-point scale (from extremely weak to extremely strong) asking “How strong are your family’s religious beliefs or practices?”.

Urban background is a dummy variable indicating that the respondent would characterize the place where they grew up as urban as opposed to rural.

### Analysis

The data processing and analyses were carried out using R 3.4.3 (R Core Team, 2017).

We used linear mixed effect (LME) models including random intercepts for family first, to find out the most important discipline types in terms of emotional processing of the child and second, to examine whether these discipline types moderated the effect of maternal traumatic stress on children’s emotional processing. We used generalized linear models (GLM, logistic regression) to examine which personal and contextual factors are associated with the use of these discipline types by mothers.

In the first LME model, seven different discipline types were entered in the model together with child demographics and maternal traumatic stress as independent variables, and the emotional processing score as the dependent variable. In the second LME model, the variables showing significance in first regression model were retained (Spanking, Take Away Media, Parent stress, Age) and all 2-way interactions were also included in the model. The emotional processing score served as the dependent variable. Finally, the GLM-models were constructed by entering mother’s age, education, religiosity, urban background, migration year, downward mobility and Discrimination Index as independent variables, and the use of discipline type (yes/no) as dependent variables.

## Results

### Descriptives

Traumatic stress scores of mothers averaged 18.4 (SD = 5.4, range = [6, 28], with a cutoff for PTS at 14 points). Based on this cutoff, it is estimated that 81% of mothers in our sample suffer from post-traumatic stress disorder (PTSD) (based on thresholds for PCL-C_Abbreviated_). Mothers used multiple methods to keep discipline. Almost all mothers (98%) used Talking with a child about his/her actions, more than half (60%) used taking away TV/Phone, Game or Activity privileges, less than half used Grounding (43%) and Spanking (42%), 38% used Sending a child to his/her room for 1 h or more, 28% used Household Chores/Duties and 9% used Putting a child in a short time out (less than 1 h). Children performed the emotional processing task well. On average, children identified 62% of emotions correctly. The descriptive statistics of demographics, maternal stress, and discipline types are presented in Table 1.

**Table 1:** Descriptives

	*N*	Missing	Mean	Median	SD	Minimum	Maximum
Emotional processing	212	0	0.62	0.64	0.11	0.16	0.81
Age	212	0	12.24	12.00	3.08	6	18
Sex	212	0	0.44	0.00	0.50	0	1
PTS	208	4	18.36	18.00	5.41	6	28
Grounding	208	4	0.41	0.00	0.49	0	1
Spanking	208	4	0.42	0.00	0.49	0	1
Chores	208	4	0.28	0.00	0.45	0	1
Send to room 1 h +	208	4	0.38	0.00	0.49	0	1
Take Away Media	208	4	0.60	1.00	0.49	0	1
Timeout (< 1 h)	208	4	0.09	0.00	0.28	0	1
Talk with child	208	4	0.98	1.00	0.15	0	1

### Maternal traumatic stress, discipline types, and emotional processing

We first examine which discipline types are associated with children’s emotional processing by including all seven discipline types in the model, together with demographics (child age and sex) and maternal stress (Model 1). The analysis showed that only Spanking ((*B*) = − 0.04, 95% CI = [− 0.07, − 0.00]) and Taking away media ((*B*) = 0.04, 95% CI = [0.00, 0.07]) contributed to children’s emotional processing, in addition to maternal PTS ((B) =  − 0.00, 95% CI = [− 0.01, − 8.37e − 4]) and child’s age ((*B*) = 0.01, 95% CI = [0.01, 0.01) (Table 2.) The results suggest that children whose mothers scored high in PTS showed poorer emotional processing and that older children in general scored higher in this task than younger children (as expected based on [11]). The results, controlling for PTS, also mean that children whose mothers used Spanking as a discipline type performed poorer in emotion processing task than children whose mothers did not used this discipline type, and that children whose mothers used Taking away of media performed better on the emotional processing task than children whose mothers did not used this discipline type.

**Table 2:** Linear mixed effects model of demographics, maternal PTS and discipline types on emotional processing of a child (Model 1)

	Estimate	SE	95% Confidence interval		*df*	*t*	*p*
			Lower	Upper			
(Intercept)	0.58	0.03	0.52	0.65	85.06	17.67	< .001
Child age	0.01	0.00	0.01	0.01	180.86	4.62	< .001
Child sex	0.01	0.01	− 0.02	0.03	187.99	0.69	0.491
Mother’s PTS	− 0.00	0.00	− 0.01	− 8.37e-4	93.06	− 2.48	0.015
Grounding	0.01	0.02	− 0.03	0.04	90.17	0.30	0.765
Spanking	− 0.04	0.02	− 0.07	− 0.00	93.21	− 2.22	0.029
Chores	− 0.03	0.02	− 0.06	0.01	94.19	− 1.32	0.191
Send to room	− 0.01	0.02	− 0.04	0.03	90.62	− 0.49	0.624
Take Away Media	0.04	0.02	0.00	0.07	89.74	2.09	0.040
Timeout	− 0.02	0.03	− 0.08	0.04	94.39	− 0.72	0.476
Talk with child	0.05	0.06	− 0.06	0.16	82.83	0.94	0.348

These two discipline types were then carried over to the next model (Models 2–7) together with maternal stress, the child’s age and their two-way interactions as separate fixed effects to assess whether these discipline types moderate the impact of maternal stress on emotional processing (Table S1). However, none of these interactions was statistically significant, meaning that discipline types did not moderate the effect of maternal stress on emotional processing of the child.

### Maternal factors associated with the use of discipline types

Since Spanking and Taking away of media as discipline types seemed to play an independent role in children’s emotional processing, we explored which personal and community related factors are related to the maternal use of these discipline types (Tables 3 and 4).

**Table 3:** Logistic regression model of background characteristics on maternal use of Spanking

Names	Estimate	SE	exp(*B*)	95% Exp(B) confidence interval		*z*	*p*
				Lower	Upper		
(Intercept)	− 0.35	0.36	0.71	0.34	1.42	− 0.96	0.336
Age	− 1.16	0.34	0.31	0.15	0.58	− 3.42	< .001
Education	− 0.14	0.33	0.87	0.44	1.65	− 0.42	0.677
Religiosity	− 0.82	0.31	0.44	0.23	0.79	− 2.61	0.009
Urban background	− 1.32	0.74	0.27	0.06	1.07	− 1.78	0.075
Migration year	− 0.13	0.29	0.88	0.48	1.54	− 0.46	0.649
Downward mobility	0.78	0.34	2.17	1.16	4.50	2.28	0.023
Discrimination Index	1.10	0.33	2.99	1.64	6.10	3.31	< .001

Regarding Spanking, the model showed that younger mothers were 2.6 times more prone to use Spanking than older mothers ((exp*B*) = 0.339, 95% CI = [0.1502, 0.577]). Less religious mothers were 1.1. times more prone to use Spanking than those who scored higher in religiosity ((*B*) = 0.440, 95% CI = [0.1502, 0.577]). In addition, mothers who have more experiences of downward mobility and discrimination were 1.7 times and 2.3 times more prone to use Spanking than mothers who scored low on these experiences ((expB) = 2.175, 95% CI = [1.1603, 4.501]) ((exp(*B*) = 2.993, 95% CI = [1.6352, 6.105]) (Table 3). Regarding Taking away media, the model revealed no significant association (Table 4).

**Table 4:** Logistic regression model of background characteristics on Taking away the media

Names	Estimate	SE	exp(*B*)	95% Exp(B) confidence interval		z	*p*
				Lower	Upper Upper		
(Intercept)	0.7366	0.324	2.089	1.133	4.086	2.2721	0.023
Age	− 0.7509	0.285	0.472	0.259	0.800	− 2.6344	0.008
Education	0.3559	0.277	1.427	0.846	2.541	1.2851	0.199
Religiosity	0.2734	0.252	1.314	0.809	2.193	1.0856	0.278
Urban background	0.1547	0.567	1.167	0.382	3.599	0.2729	0.785
Migration year	0.2542	0.267	1.289	0.773	2.240	0.9517	0.341
Downward mobility	-0.0203	0.268	0.980	0.573	1.662	-0.0756	0.940
Discrimination index	0.3090	0.252	1.362	0.839	2.273	1.2262	0.220

## Discussion

The ability to perceive and reason about other people’s emotions is an essential adaptive skill. Everyday interactions between individuals is largely dependent on making reasonable inferences about how other people may be feeling or reacting. Children who perform poorly in this are likely to have social and interpersonal difficulties.

With this paper, we aimed to shed light on families that experience a heavy everyday burden living in communities outside refugee camps in a country neighboring that of their origin. As a consequence of their experiences, many refugees struggle with war-related mental health problems. Parental mental health together with everyday struggle affect the intergenerational effects of stress produced by armed conflict. Under the stress, parenting practices may change and harsh parenting has been found to be more common in these circumstances [3, 6]. This study showed that both maternal wellbeing and parenting practices have an important role in shaping the emotional processing in children.

The use of certain type of discipline did not seem to strengthen or alleviate the effect of maternal trauma symptoms on child emotional processing capacity. However, nearly half of the mothers used Spanking as a discipline, and that was directly associated with poorer emotional processing capacity of a child, putting the next generation in the risk of mental health problems. In contrast, children whose mothers limited their access to media as a way of keeping the discipline, performed better on the emotional processing task.

The lack of interaction effect between maternal trauma and discipline type on emotional processing of a child reflects the independent role of mental health and behavior of a mother on child’s cognition. However, future studies with larger samples should enable the search for the interaction effects between different levels of symptom severity and harsh parenting. It is possible that the extreme forms of traumatization and severe or frequent use of violent parenting practices could lead to added negative effects on child emotional processing, especially related to fear and other negative emotions.

Our study contradicts the earlier findings of harsh parenting and its effects on child emotional processing, suggesting that Spanking as a discipline style is indeed associated with lower performance in emotional processing task [21–23]. It is noteworthy that the current study was conducted in a different population (western vs. middle eastern), under different circumstances (peace- vs. war time), and using different methods (sentences and drawings vs. images of real faces) than the earlier studies. These factors may have contributed to different findings compared to earlier studies. On the other hand, the results are in line with the recent meta-analysis showing that early adversity, in general, is associated with a lower accuracy rate on face recognition, especially concerning fearful and happy faces [20].

It was not possible to examine the effects of different types of emotion (e.g., anger, sadness) in our study, so it would be important to replicate the study in a larger sample and to detect the possible differences in recognition of different emotions in the context of harsh parenting. In addition, some possibly important parenting practices, such as neglect, maltreatment or rewarding good behavior, were out of the scope of this study. Earlier studies show that war time atrocities and stress affect parent–child relationship and parenting practices [3, 6, 19]. In our study, the prevalence of maternal PTS was high, and including the broader spectrum of parenting factors in future studies may help in completing the picture of the effects of parenting on child emotional processing.

Children’s access to media has rapidly grown. To our knowledge, no studies so far have evaluated its effects on emotional processing using face recognition task, at least not in refugee population. This finding needs further elaboration and can indeed be an important factor to consider in parent training programs, also in vulnerable populations.

The age of a child affects emotional processing of a child. Most studies show that by age 5–6 years, most children accurately recognize and label static images of facial configurations on tasks similar to the one used here similarly to adults [39]. However, on the broader issue of how humans construe and understand emotions, there are significant changes in conceptual understanding between 6 year olds and adults [40]. The earlier research, using the same data that in this study, showed that for each year child became older, the odds of correctly identifying an emotion became 1.05 times larger [30]. It is noteworthy that we did not find an interaction effect between child’s age and discipline types on child emotional processing, meaning that the effect of parenting, maybe even surprisingly, affects the same way among younger and older children. The finding implies that the effect of harsh parenting (for example) on emotional processing of a child is not age sensitive but causes a risk on poor processing even if child is already a teenager.

Socio-cultural factors influence parenting practices, and ignoring these factors can lead to overgeneralizations about parenting [41, 42]. In this study, we wanted to know which factors put mothers at risk of using a negative discipline style such as Spanking. In line with earlier studies [24, 25], the profile of parents whose children showed poor emotional processing seems to reflect an already underprivileged group, young mothers with downward mobility, that have experienced discrimination and low religiousness (possibly affecting on social activity in the form of social gatherings and support). At the same time, none of the factors we studied was associated with maternal use of Taking away media as a discipline strategy. Finding out which characteristics underlie positive parenting practices is crucial. This would enable boosting the conditions under which parents will have the best resources to support their child’s cognitive development. The information is urgently needed in refugee settings and also in other contexts where parenthood is difficult.

The unique role of mothers in this sample suggests a higher vulnerability for their children and should be taken into account in future interventions. First, improving the human rights of mothers can have far reaching benefits, as it can diminish the use of harsh parenting practices and subsequently enhance the optimal emotional processing of a child. Making lives easier for mothers might be more effective, and have larger positive effects on the development of their children. Second, treating the trauma of parents is important. Effective treatments exist and have been demonstrated to alleviate symptoms in 60–80% of cases [43]. However, refugees, often due to co-morbidity, do not always reach optimal levels of treatment effects [44]. To tackle the negative intergenerational effects, parenting programs are also needed. It is possible that the current results can be generalized to other contexts where mothers experience hardship related to their own mental health problems as well as the lack of other resources that can support them and their families.

## Limitations

In a cross-sectional study like ours, we have to consider the fact that poor emotional processing of a child may affect parenting practices. Poor emotional processing is highly correlated with other problems in emotion development, which in turn challenges the interaction between a child and a parent [45]. However, it is unlikely that parents’ PTSs are caused by children’s poor emotional processing, so the child is not the sole driver in the context described in this paper. Profile of mothers that use Spanking also suggest that there are other, non-child related, factors that impact on this. Longitudinal studies are needed to find out the developmental pathways between parental wellbeing, parenting practices and child’s cognitive capacity.

Although most refugees from Syria do not live in refugee camps, there are no registries of people living in Turkish communities. To collect the data from these families, we were forced to rely on an opportunistic sampling procedure where each family helped in recruiting other participants. The registry of refugees would have been easier to achieve in refugee camps but it would have been difficult to generalize the results to other settings. Using multiple settings, both community-based and random, representative sample of families in camps is needed in future.

Unfortunately, due to low participation of fathers, we could only run the quite complicated analysis among mothers. Therefore, we do not know whether fathers’ choice of discipline would have been different or have a different effect? A future study could look at this using two independent or interactive moderators of mothers and fathers discipline types.

Finally, all questionnaires and instructions were translated into Arabic, and back-translated to English. The standardized Arabic versions of all tests (including databases of emotional faces from the region) were unfortunately not available, representing another area of improvement for future studies.

## Supplementary Information

Below is the link to the electronic supplementary material.

Supplementary file1 (DOCX 30 KB)
