# Supplementary material for: The role of maternal trauma and discipline types in emotional processing among Syrian refugee children
Source: Eur Child Adolesc Psychiatry. 2022 Feb 26;32(8):1487–95. doi: 10.1007/s00787-022-01962-3 (PMC10326120; doi:10.1007/s00787-022-01962-3)
Supplement: Supplementary file 1 — Supplementary file1 (DOCX 30 KB) [file 787_2022_1962_MOESM1_ESM.docx]

| *Table S1. Linear Mixed Effects Models of Demographics, Maternal PTS, two Discipline types and their interactions on Emotional Processing of a Child (Models 2-7.)* | | | | | | | | | | | | | |  | |  | |  | |  | |  | |  | |
| --- | --- | --- | --- | --- | --- | --- | --- | --- | --- | --- | --- | --- | --- | --- | --- | --- | --- | --- | --- | --- | --- | --- | --- | --- | --- |
|  | | | | | | | | | | | | | | | | | | | | | | | | | |
|  | | | | | | | | | **95% Confidence Interval** | | | | |  | | | | | | | | | | | |
| **Names** | | **Effect** | | **Estimate** | | **SE** | | | **Lower** | | **Upper** | | | **df** | | | | **t** | | | | **p** | | | |
| **Model 2** | | | | | | | | | | | | | | | | | | | | | | | | | |
| (Intercept) |  | (Intercept) |  | 0.62 |  | 0.01 |  | 0.61 | |  | 0.64 |  | 84.71 | |  | | 69.69 | |  | | < .001 | |  | |  |
| Age |  | Age |  | 0.01 |  | 0.00 |  | 0.01 | |  | 0.01 |  | 179.48 | |  | | 4.76 | |  | | < .001 | |  | |  |
| PTS |  | PTS |  | -0.00 |  | 0.00 |  | -0.01 | |  | -8.94e−4 |  | 91.56 | |  | | -2.52 | |  | | 0.013 | |  | |  |
| Spanking1 |  | 1 - 0 |  | -0.03 |  | 0.02 |  | -0.07 | |  | 0.00 |  | 88.03 | |  | | -1.86 | |  | | 0.066 | |  | |  |
| Take Away Media1 |  | 1 - 0 |  | 0.03 |  | 0.02 |  | -0.00 | |  | 0.07 |  | 86.31 | |  | | 1.68 | |  | | 0.096 | |  | |  |
| Spanking1 ✻ Take Away Media1 |  | 1 - 0 ✻ 1 - 0 |  | -0.03 |  | 0.04 |  | -0.10 | |  | 0.04 |  | 85.31 | |  | | -0.78 | |  | | 0.438 | |  | |  |
| **Model 3** | | | | | | | | | | | | | | | | | | | | | | | | | |
| (Intercept) |  | (Intercept) |  | 0.63 |  | 0.01 |  | 0.61 | |  | 0.64 |  | 88.48 | |  | | 69.10 | |  | | < .001 | |  | |  |
| Age |  | Age |  | 0.01 |  | 0.00 |  | 0.01 | |  | 0.01 |  | 178.55 | |  | | 4.92 | |  | | < .001 | |  | |  |
| PTS |  | PTS |  | -0.00 |  | 0.00 |  | -0.01 | |  | -0.00 |  | 92.61 | |  | | -2.62 | |  | | 0.010 | |  | |  |
| Spanking1 |  | 1 - 0 |  | -0.04 |  | 0.02 |  | -0.07 | |  | -7.32e−4 |  | 93.29 | |  | | -2.00 | |  | | 0.048 | |  | |  |
| Take Away Media1 |  | 1 - 0 |  | 0.03 |  | 0.02 |  | -0.00 | |  | 0.07 |  | 89.62 | |  | | 1.72 | |  | | 0.089 | |  | |  |
| Age ✻ Spanking1 |  | Age ✻ 1 - 0 |  | 0.01 |  | 0.00 |  | -0.00 | |  | 0.02 |  | 179.69 | |  | | 1.69 | |  | | 0.092 | |  | |  |
| **Model 4** | | | | | | | | | | | | | | | | | | | | | | | | | |
| (Intercept) |  | (Intercept) |  | 0.62 |  | 0.01 |  | 0.61 | |  | 0.64 |  | 85.33 | |  | | 68.98 | |  | | < .001 | |  | |  |
| Age |  | Age |  | 0.01 |  | 0.00 |  | 0.01 | |  | 0.01 |  | 173.67 | |  | | 4.56 | |  | | < .001 | |  | |  |
| PTS |  | PTS |  | -0.00 |  | 0.00 |  | -0.01 | |  | -9.20e−4 |  | 91.59 | |  | | -2.54 | |  | | 0.013 | |  | |  |
| Spanking1 |  | 1 - 0 |  | -0.04 |  | 0.02 |  | -0.07 | |  | -0.00 |  | 93.24 | |  | | -2.11 | |  | | 0.037 | |  | |  |
| Take Away Media1 |  | 1 - 0 |  | 0.03 |  | 0.02 |  | -0.00 | |  | 0.07 |  | 88.85 | |  | | 1.82 | |  | | 0.072 | |  | |  |
| Age ✻ Take Away Media1 |  | Age ✻ 1 - 0 |  | 5.26e-4 |  | 0.00 |  | -0.01 | |  | 0.01 |  | 178.56 | |  | | 0.12 | |  | | 0.901 | |  | |  |
| **Model 5** | | | | | | | | | | | | | | | | | | | | | | | | | |
| (Intercept) |  | (Intercept) |  | 0.62 |  | 0.01 |  | 0.61 | |  | 0.64 |  | 83.81 | |  | | 69.54 | |  | | < .001 | |  | |  |
| Age |  | Age |  | 0.01 |  | 0.00 |  | 0.01 | |  | 0.01 |  | 179.73 | |  | | 4.76 | |  | | < .001 | |  | |  |
| PTS |  | PTS |  | -0.00 |  | 0.00 |  | -0.01 | |  | -0.00 |  | 91.07 | |  | | -2.62 | |  | | 0.010 | |  | |  |
| Spanking1 |  | 1 - 0 |  | -0.04 |  | 0.02 |  | -0.07 | |  | -0.00 |  | 91.98 | |  | | -2.08 | |  | | 0.041 | |  | |  |
| Take Away Media1 |  | 1 - 0 |  | 0.03 |  | 0.02 |  | -0.00 | |  | 0.07 |  | 88.16 | |  | | 1.87 | |  | | 0.065 | |  | |  |
| PTS ✻ Spanking1 |  | PTS ✻ 1 - 0 |  | -0.00 |  | 0.00 |  | -0.01 | |  | 0.00 |  | 90.74 | |  | | -0.73 | |  | | 0.466 | |  | |  |
| **Model 6** | | | | | | | | | | | | | | | | | | | | | | | | | |
| (Intercept) |  | (Intercept) |  | 0.62 |  | 0.01 |  | 0.61 | |  | 0.64 |  | 86.22 | |  | | 70.08 | |  | | < .001 | |  | |  |
| Age |  | Age |  | 0.01 |  | 0.00 |  | 0.01 | |  | 0.01 |  | 181.01 | |  | | 4.77 | |  | | < .001 | |  | |  |
| PTS |  | PTS |  | -0.00 |  | 0.00 |  | -0.01 | |  | -3.32e−4 |  | 88.11 | |  | | -2.17 | |  | | 0.033 | |  | |  |
| Spanking1 |  | 1 - 0 |  | -0.04 |  | 0.02 |  | -0.07 | |  | -0.00 |  | 93.88 | |  | | -2.06 | |  | | 0.042 | |  | |  |
| Take Away Media1 |  | 1 - 0 |  | 0.03 |  | 0.02 |  | -0.00 | |  | 0.07 |  | 89.82 | |  | | 1.85 | |  | | 0.068 | |  | |  |
| PTS ✻ Take Away Media1 |  | PTS ✻ 1 - 0 |  | -0.01 |  | 0.00 |  | -0.01 | |  | 0.00 |  | 88.07 | |  | | -1.57 | |  | | 0.120 | |  | |  |
| **Model 7** | | | | | | | | | | | | | | | | | | | | | | | | | |
| (Intercept) |  | (Intercept) |  | 0.62 |  | 0.01 |  | 0.61 | |  | 0.64 |  | 82.19 | |  | | 69.26 | |  | | < .001 | |  | |  |
| Age |  | Age |  | 0.01 |  | 0.00 |  | 0.01 | |  | 0.01 |  | 178.50 | |  | | 4.72 | |  | | < .001 | |  | |  |
| PTS |  | PTS |  | -0.00 |  | 0.00 |  | -0.01 | |  | -9.16e−4 |  | 89.05 | |  | | -2.53 | |  | | 0.013 | |  | |  |
| Spanking1 |  | 1 - 0 |  | -0.04 |  | 0.02 |  | -0.07 | |  | -0.00 |  | 89.90 | |  | | -2.10 | |  | | 0.038 | |  | |  |
| Take Away Media1 |  | 1 - 0 |  | 0.03 |  | 0.02 |  | -0.00 | |  | 0.07 |  | 85.82 | |  | | 1.83 | |  | | 0.071 | |  | |  |
| Age ✻ PTS |  | Age ✻ PTS |  | -2.25e−5 |  | 3.60e-4 |  | -7.28e−4 | |  | 6.83e-4 |  | 177.77 | |  | | -0.06 | |  | | 0.950 | |  | |  |
| Notes: Linear mixed effect (LME) models including random intercepts for family. Coding for categorical variables defined in R using the contr.treatment(k)-(1/k). In models that include interaction terms, the other variable effects are computed averaging across the sample. | | | | | | | | | | | | | | | | | | | | | | | | | |
